# Improved Continuum Joint Configuration Estimation Using a Linear Combination of Length Measurements and Optimization of Sensor Placement

**DOI:** 10.3389/frobt.2021.637301

**Published:** 2021-04-01

**Authors:** Levi Rupert, Timothy Duggan, Marc D. Killpack

**Affiliations:** ^1^Robotics and Dynamics Lab, Department of Mechanical Engineering, Brigham Young University, Provo, UT, United States; ^2^Otherlab Inc., San Francisco, CA, United States

**Keywords:** estimation, optimization, continuum joints, soft robotics, proprioception

## Abstract

This paper presents methods for placing length sensors on a soft continuum robot joint as well as a novel configuration estimation method that drastically minimizes configuration estimation error. The methods utilized for placing sensors along the length of the joint include a single joint length sensor, sensors lined end-to-end, sensors that overlap according to a heuristic, and sensors that are placed by an optimization that we describe in this paper. The methods of configuration estimation include directly relating sensor length to a segment of the joint's angle, using an equal weighting of overlapping sensors that cover a joint segment, and using a weighted linear combination of all sensors on the continuum joint. The weights for the linear combination method are determined using robust linear regression. Using a kinematic simulation we show that placing three or more overlapping sensors and estimating the configuration with a linear combination of sensors resulted in a median error of 0.026% of the max range of motion or less. This is over a 500 times improvement as compared to using a single sensor to estimate the joint configuration. This error was computed across 80 simulated robots of different lengths and ranges of motion. We also found that the fully optimized sensor placement performed only marginally better than the placement of sensors according to the heuristic. This suggests that the use of a linear combination of sensors, with weights found using linear regression is more important than the placement of the overlapping sensors. Further, using the heuristic significantly simplifies the application of these techniques when designing for hardware.

## 1. Introduction

Continuum joints are becoming a common style of robotic joint, especially in the world of soft robotics. These joints bend continuously along their length and offer the ability to form complicated shapes, operate in cluttered environments, and can be compliant which increases the inherent safety of the robot.

**Figure 1:**
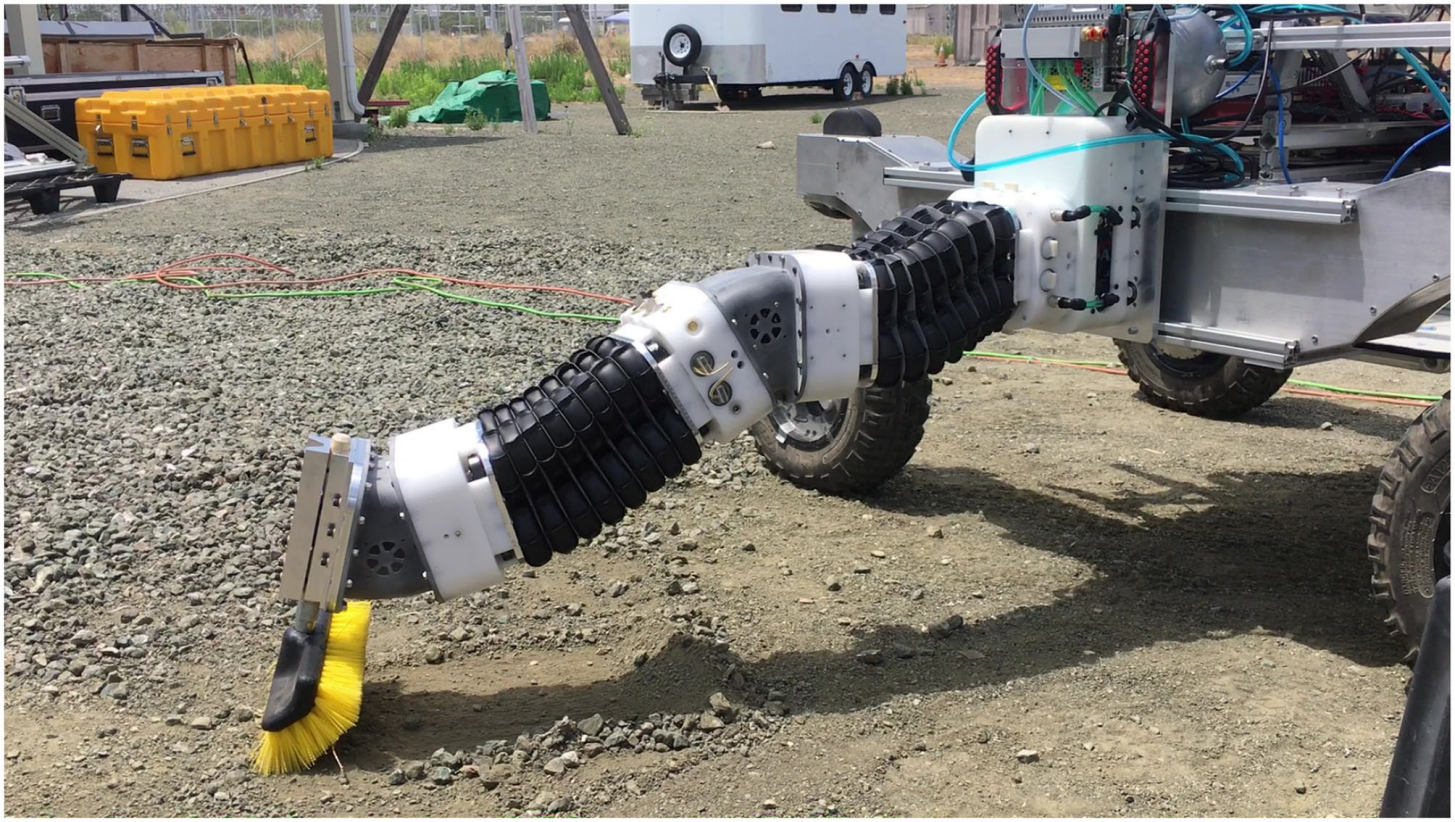
A compliant continuum robot exhibiting non-constant curvature bending in its joints (especially in the first joint) when under load while performing a real-world task.

While being able to form complicated shapes and easily deform is one of continuum joints biggest strengths, it is also one of the attributes that make them the hardest to use in practice. Current approaches for sensing the configuration of continuum robots include many different methods such as motion capture, optical sensors, and length sensors (see section 1.1 for a review of many of the methods used for sensing continuum joint's state). Many of these state-of-the-art methods operate under assumptions that limit their ability to estimate the full kinematic position of a continuum joint in non-laboratory settings, (e.g., settings where the joint undergoes actual loads during a useful task that cause unanticipated bending). In this work we focus on methods that use measurements of the length of a continuum joint to estimate the configuration of the joint. Many of the previous methods in the literature assume that the bending of the joint is constant curvature. This assumption readily breaks down as soon as any actual loads are applied to the joint. Of particular note, is when the robot is in the s-shape bending as shown in Figure 1 (see proximal joint). For any method using a single length measurement and a constant curvature assumption, the measurement in this scenario will result in an estimate of zero deflection.

The most accurate way to sense the full configuration of a continuum joint using length sensors, would be to divide the joint into infinitesimal segments and have each of those segments be monitored by length sensors. For every sensor that is added to the joint, the constant curvature assumption can be applied to that smaller segment. By having every segment covered by its own sensor the full configuration could be reconstructed by treating those segments as pieces of a kinematic chain.

This method is not feasible for a real system due to mechanical, electrical, and computational limits. However, it does suggest that it may be possible to increase the number of sensors to get more accurate pose estimation of a continuum joint while still remaining within the mechanical, electrical, and computational constraints of a real system.

However, if sensors are simply placed end to end along the joint, estimation will still be limited to the maximum number of sensors that will be allowed by the physical constraints of the system. Our hypothesis was that by using measurements from sensors that have overlapping coverage of the same discrete joint segments, whether literal discrete segments like the robot shown in Figure 1, or representative segments of a soft robot, a more accurate estimate can be accomplished. In this paper, we show that by overlapping sensors on the joint, more information can be gained without the cost of adding a sensor for every representative segment.

We propose and demonstrate two new methods of estimating the configuration of a continuum joint using measurements from overlapping length sensors. The first method averages the per segment length of the sensors for each sensor monitoring a segment, we call this the Equally-Weighted Averaging Method (E-WAM). The second method estimates the segment configuration by using a linear combination of the per segment lengths of all the sensors on the continuum joint, we call this the Weighted Averaging Method (WAM). The weights for the WAM method are found by performing a linear regression as discussed in section 2.5.

To determine the placement of the overlapping sensors, we developed a heuristic placement method as well as an Evolutionary Algorithm (EA) that determines optimal sensor placement for a joint. We compare the results of these two placement methods in this paper.

The primary contributions of this paper are:

1. The novel concept of overlapping length sensors to improve the estimation of a continuum joint's state.
2. WAM: A method for using overlapping sensors to significantly improve continuum joint estimation resulting in an estimate that reduces error by a factor of eleven when using two sensors on a joint rather than using a single sensor.
3. Two methods for determining the placement of overlapping length sensors on a continuum joint, and an objective comparison of their performance.

All of the methods and theory that we develop in this paper are based purely on kinematics and static loading conditions. We confirm and demonstrate our contributions using a Piece-Wise Constant Curvature continuum joint kinematic simulation. Future work would include implementing this on actual hardware and in dynamic environments.

The remainder of this paper is organized as follows, section 1.1 discusses related literature on sensor design and estimation for continuum actuators, as well as methods for determining optimal sensor placement. Section 2 discusses the assumptions we used and develops the models, theory, and algorithms for our estimation methods. Section 3 presents the results of the estimations methods and section 4 discusses the results and possible applications for future work.

### 1.1. Related Work

The focus of this paper is estimation for a discrete-segment continuum joint. Although there are many types of soft robot joints [including discrete segments (Hannan and Walker, 2003), compliant continuum joints with discrete rigid components (Rone and Ben-tzvi, 2013), and fully soft-bodied robots (Marchese et al., 2014)], we have chosen to develop our methods for discrete segments because (1) it matches our actual development hardware, and (2) most soft robot joints could be represented to varying degrees of fidelity by a discrete-segment model where the kinematics are approximated with a series of representative constant curvature segments, regardless of actual construction.

Related literature can be divided into two main areas: (1) soft robot configuration estimation; (2) soft robot sensor placement.

#### 1.1.1. Soft Robot Configuration Estimation

Of the two areas covered in this paper, by far the most literature exists relative to novel sensors for soft robot configuration estimation. We therefore describe prior work that uses different methods of construction or physical phenomenon to estimate soft robot configuration. We also describe methods used to estimate the actual bend angle or pose.

A significant amount of the research in soft robot configuration estimation has required using motion capture systems with infrared cameras and reflective tracking dots (Marchese et al., 2014; Katzschmann et al., 2019), electro-magnetic field detectors (Song et al., 2015; Anderson et al., 2017; Gerboni et al., 2017), or virtual reality tracking hardware (Hyatt et al., 2019). However, using this type of sensor constrains the mobility of the soft robot to operate solely within the range of the motion capture system.

Resistance-based sensing is often used with conductive material or fabrics that are assembled in a way such that the resistance of a circuit varies as the bend angle of the robot changes. Examples use methods ranging from commercial flex sensors (Ozel et al., 2016), to conductive thread (Cianchetti et al., 2012; Zhao and Abbas, 2016; Abbas and Zhao, 2017), or yarn (Wurdemann et al., 2015), to conductive silicone that is cut using principles from kirigami (Truby et al., 2020). There are multiple examples of this approach (see Gibbs and Asada, 2005; She et al., 2015; Elgeneidy et al., 2016, 2018; Yuen et al., 2017; Zhou et al., 2020).

Many papers have focused on using optical methods that tend to revolve around novel combinations or topologies for Fiber Bragg Grating (FBG) sensors (see Wang et al., 2016; Zhuang et al., 2018; He et al., 2019; Sheng et al., 2019). However, other related methods focus on the basic idea of using optical fibers in general (see Yuan et al., 2011; Chen et al., 2019; Godaba et al., 2020). Using optical frequency domain reflectometry combined with added optical gratings the authors in Monet et al. (2020) were able to show that they could improve configuration estimation when in contact or with non-constant curvature for medical applications.

Some methods have relied on photo diodes (Dobrzynski et al., 2011), or combined the strength of traditional camera or ultrasound images in conjunction with optical fibers (see Denasi et al., 2018; Wang et al., 2020). Other researchers used camera-based methods directly to detect contact, or estimate deformation for a deformable link, but with rigid joints (Oliveira et al., 2020).

Other physical phenomenon used include capacitance (Yuen et al., 2017, 2018; Bilodeau et al., 2018; Case et al., 2018), inductance (Felt et al., 2016, 2018, 2019), magnetism (Ozel et al., 2016), impedance (Avery et al., 2019), or a combination of gyroscope, accelerometer, and magnetometer in an inertial measurement unit (IMU) package (Hyatt et al., 2019).

Similar to our efforts to include multiple sensors to improve configuration estimation, there are some researchers who have used overlapping sensors to improve performance. Specifically, Li et al. (2020) used a dual array FBG scheme to improve estimation accuracy. While Felt et al. (2019) used two circuits and measured change in inductance to improve estimation of lateral motion for a continuum joint.

As near as we can tell, all of the previous sensors and estimation methods (minus those that give a global pose such as motion capture) seem to focus on estimating curvature *or* linear motion only, which does not account for deformation that we would expect when these platforms are heavily loaded. Some methods enable detection of contact, but this is used as a way to relate discrepancies in curvature to a contact event, rather than using the loading condition to more accurately estimate the joint configuration with a non-constant curvature assumption. However, there is some literature where the configuration of flexible members experiencing a point load is estimated using accurate Kirchoff or Cosserat rod models and additional sensor information (such as cameras or force-torque data). In Rucker and Webster (2011) they use an Extended Kalman Filter in conjunction with a Cosserat rod model which requires a measurement of the tip pose and applied forces. While in Borum et al. (2014) the authors use external cameras to help solve for the configuration of a flexible member that can have multiple equilibrium positions (due to bifurcation) by formulating the problem as a geometric optimal control solution. This solution includes estimates for the forces and torques applied at the tip to cause the deformation. In both cases, the deformation was restricted to being planar and was caused by an external force at the tip, rather than being included as part of a potentially self-contained soft robot control scheme.

In Trivedi and Rahn (2009) and Trivedi and Rahn (2014), the authors solve for the configuration of the OctArm robot platform with unknown payloads using Cosserat rod models and three different sensing methods (e.g., force-torque sensors and an inclinometer at the base, multiple cable encoders, and multiple inclinometers along the manipulator) to constrain and solve initial value or ODE problems with given boundary conditions. The method was effective, but required varying levels of accurate knowledge about soft robot parameters depending on the sensing method used and was again restricted to planar applications (although not due to limits in formulation). In addition, this formulation would require additional sensors across the arm if a distributed load were applied (not at the tip or end effector). Similar work uses Cosserat rod models (Sadati et al., 2020) or Kirchoff elastic rod models (Takano et al., 2017; Nakagawa and Mochiyama, 2018) combined with force sensing at the base of the flexible member in order to estimate soft robot configuration or interaction forces and stiffnesses.

These model-based methods hold great promise and could likely be incorporated with our model-free method. However, additional benefits of our method are that even without a complex soft-body model, it performs quite well and is able to handle loading conditions that are not limited to the tip of the flexible member. Any additional information derived from an accurate model within an estimation scheme such as a Kalman filter would likely improve the results shown in this paper.

Finally, using different modalities, many researchers have used neural networks to map sensor output to joint configuration for optical sensors (Sklar et al., 2016), FBG sensors plus ultrasound images (Denasi et al., 2018), pressure readings (You et al., 2017), tactile arrays (Scimeca et al., 2019), or linear potentiometers (Melingui et al., 2014; Merzouki et al., 2014; Day, 2018). In Lun et al. (2019) they develop a flexible sensor using fiber Bragg gratings that when combined with a learned model can be used to accurately reconstruct the surface of a soft robot, but this is not applied specifically to a soft robot. Many of these methods learned a mapping to estimate full pose for the tip of one, or sometimes multiple joints. However, one of the main limitations is that there is no relation or intuition between the data and the black box model that is produced. Also, if the manipulator were to carry a larger load, additional data with the load in place would likely need to be collected, especially if the joint deformed in a way that violated constant curvature assumptions. Information about the load (e.g., overall mass and distribution of mass) may also have to be included in the training data to make the approach general. Because our approach is based on fitting parameters to shapes that are caused by many different loading conditions, we expect this approach to potentially generalize more easily.

#### 1.1.2. Sensor Placement

The general problem of sensor placement (number of sensors and relative positioning) is often approached using a metric of observability in order to improve estimator design (see Krener and Ide, 2009; DeVries and Paley, 2013; Qi et al., 2015). However, observability may not always be the best metric and sensor placement based on simple models and heuristics is an open research problem (Clark et al., 2020).

For our specific contributions, we focus on sensor placement in the context of soft robot configuration estimation. Some researchers have followed the previously mentioned approach of relating soft robot sensor placement to observability (Mahoney et al., 2016). In this case they use a differential representation of the continuum robot's kinematic equations. However, the robot is a concentric tube robot which appears to be unloaded, in contrast to the work we present. In Tapia et al. (2020), they require hyperelastic material models and finite element discretizations to simulate nonlinear behavior of a given soft robot with expected loading. This is similar to our method with two main differences. Our loading and deformation models are much simpler and the proposed optimization in that paper requires the sensors to be integrated with the actual fabrication of the soft robot, unlike ours which can be added after the fact and only needs to measure length. Other relevant work includes Deutschmann et al. (2019), where the authors optimize the attachment points for length sensors to estimate the pose of a 6-DoF continuum-joint robot head. This required a beam finite element model with a fixed load (the robot head) and the data was fused with IMU. Finally, in Kim et al. (2014) they use FBG sensors and an optimization with a similar notion to our weighted reconstruction, using their own set of basis functions. However, the type of optimization presented does not necessarily translate to overlapping sensors (which we have found to be very beneficial in the results presented in this paper).

## 2. Materials and Methods

In this section we describe the methods used to develop the simulated continuum robot configuration, estimate the continuum robot configuration from the attached sensors, and develop the evolutionary algorithm used to find the optimal sensor placement along the continuum robot joint.

### 2.1. Continuum Joint Configuration

For a general continuum joint there are three degrees of freedom, bending about two orthogonal axes and extension along an axis orthogonal to the two bending axes. As long as one bending degree of freedom exists, in which the center of rotation stays constant for the bending range, the joint can be considered a continuum joint.

In our work, we focus on continuum joints that have a fixed length/height and two bending degrees of freedom. The continuum joint hardware shown in Figure 1, is used as the basis for models in this paper, is made up of bending segments of a fixed height. We assume that these segments bend with piece-wise constant curvature. The theory is that the curvature change in one segment is small enough that it can be assumed to have constant curvature. It should be noted that the methods discussed in this paper can be adapted for joints that are not actually made of smaller constant curvature segments by splitting the joint into virtual segments.

**Figure 2:**
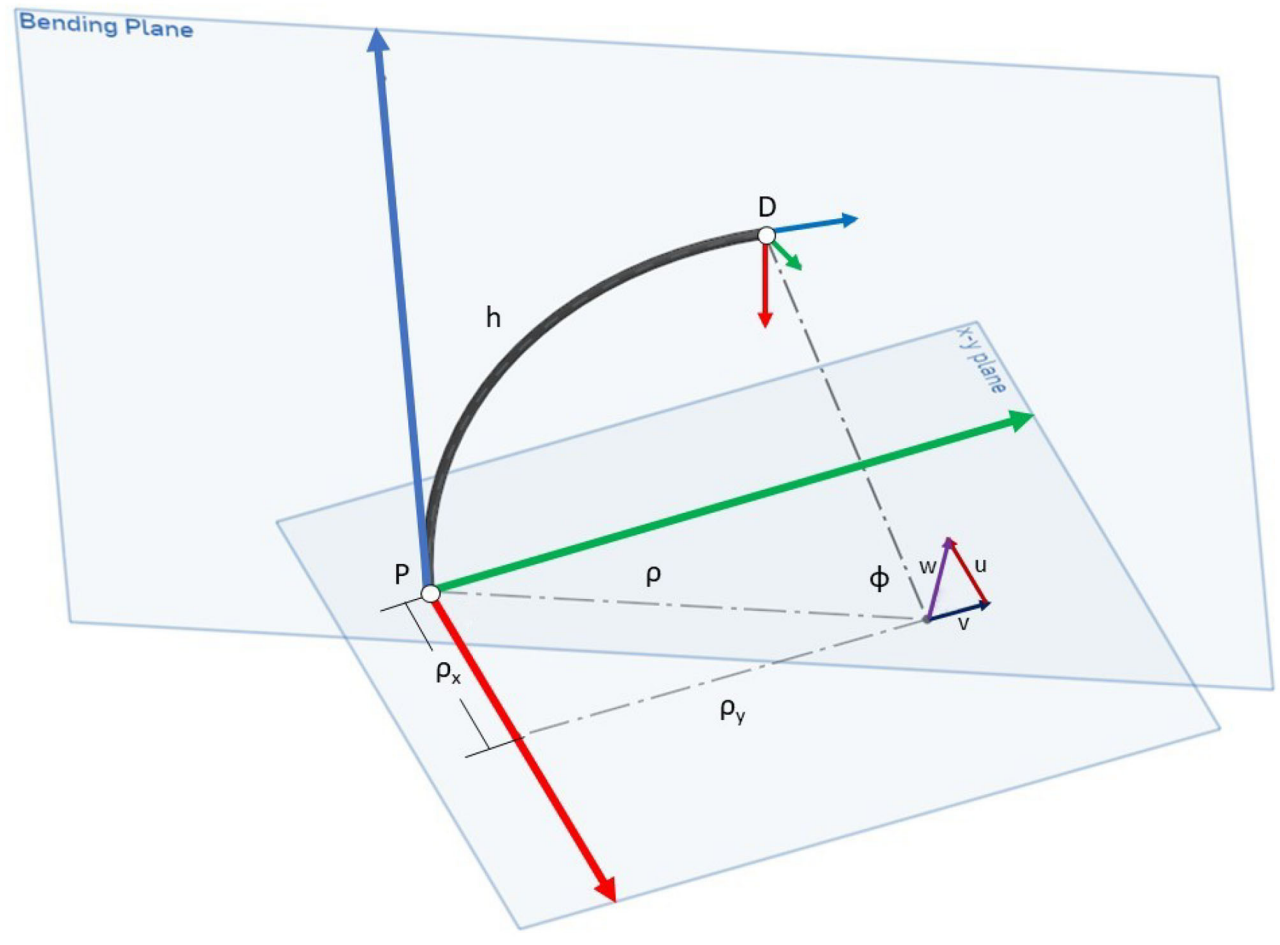
The parametrization variables used to describe a constant curvature continuum segment in our work. Developed by Allen et al. (2020).

Due to our fixed length assumption as the joint bends there exists a neutral axis or spine at the center of the joint that does not change lengths. This is represented by the black line in Figure 2.

We use the *u*, *v*, and *h* states developed by Allen et al. (2020) to describe the configuration space and pose of a single continuum joint segment under the constant curvature assumption (displayed graphically in Figure 2). This parameterization is based on Screw Theory. The full configuration of the continuum joint is described by the *u*, *v*, and *h* parameters of the series of smaller segments that make up the joint. The parameter *u* describes bending about the local x-axis and *v* describes bending about the local y-axis for each segment. The variable *h* is the length of the neutral axis which we keep constant for the purposes of this paper. According to Allen et al., the arc angle, *ϕ*, is equal to the magnitude of the rotation axis, *w* = [*u, v*, 0].

### 2.2. Sensor Arrangement

As stated previously, for this application we are simulating sensors that measure the change in length of the joint as it undergoes bending. By using constant curvature assumptions we can calculate the pose from the sensors length measurements as will be described in section 2.3.

**Figure 3:**
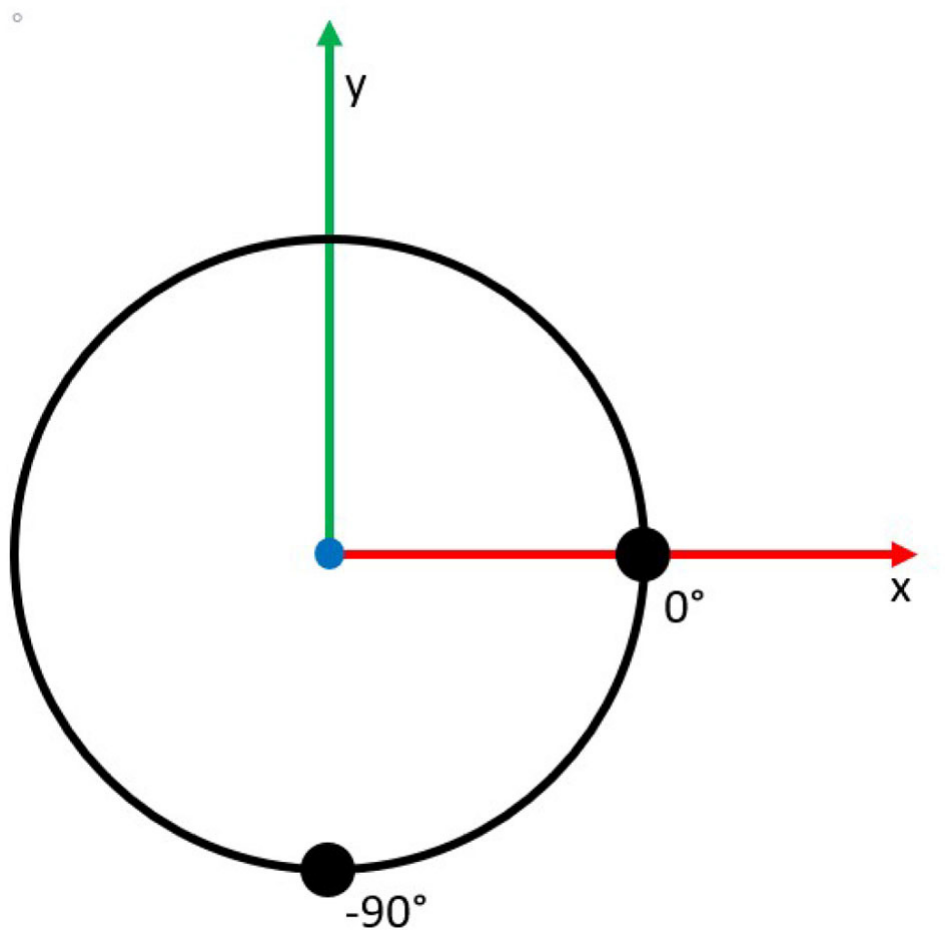
A cross-sectional illustration of the sensor locations at 0° and −90° on the circumference of the joint.

The continuum joint has two sets of sensors that run the length of the joint and start at the base at 0 and −90° on the circumference of the joint as shown in Figure 3. These locations allow each set of sensors to independently measure the two degrees of freedom (*u* and *v*) as the mounting points correspond to the directions of the bending axis. Thus, bending about each axis will only be measured by a single set of sensors.

**Figure 4:**
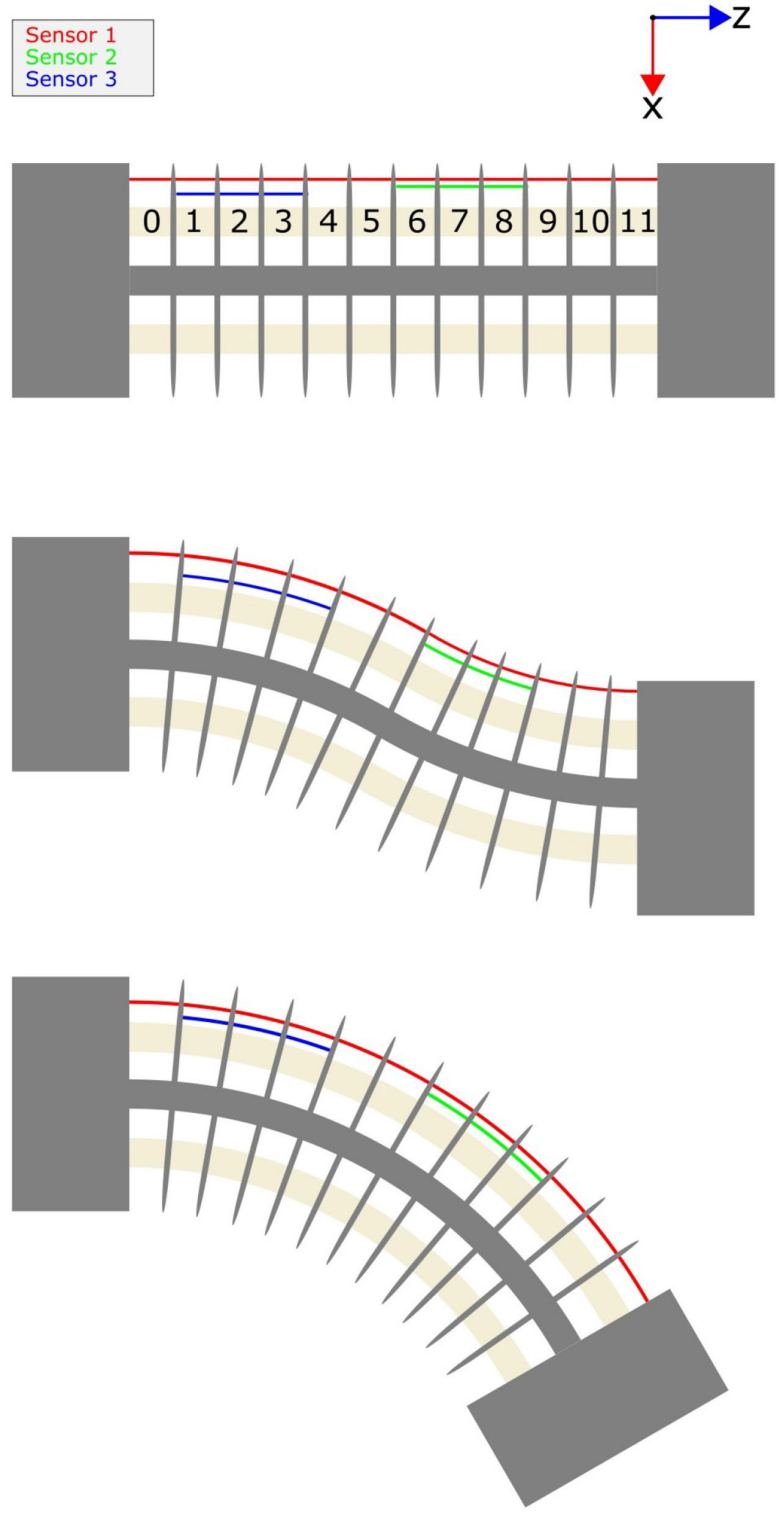
An illustration of showing three possible bending states of a continuum joint, the segment indexing used in this paper, and a possible sensor configuration.

Each set of sensors in our simulations contain between one and six sensors which are aligned such that they are parallel to the neutral axis of the continuum joint in the unbent configuration. Additionally we modeled each joint using 12–48 segments of equal length. The sensors were placed such that they cover a series of consecutive segments. This series can be a minimum of one segment or a maximum of the total number of segments representing the joint. An example of a set of three sensors is shown in Figure 4.

For a sensor configuration to be considered for our simulated experiments, each segment must be covered by a minimum of one sensor. For the joints simulated in this paper, both degrees of freedom had identical sensor placements although this is not a requirement for successful configuration estimation of a two degree of freedom continuum joint.

### 2.3. Pose Estimation

As mentioned in sections 2.1 and 2.2, the bending section of the continuum joint is divided into smaller segments that are small enough that we can assume constant curvature. Additionally each of these segments is covered by at least one length sensor located at a fixed distance away from the neutral axis.

The work developed by Allen et al. (2020) also describes how to estimate the angle of bending for a continuum joint with constant curvature that is monitored by a length sensor. We apply this method to our discrete sections by using Equations (1) and (2) which convert the length of a tendon, *l*, located at a fixed radius from the neutral axis of the joint to a joint angle, *u* or *v*, given the height of the segment, *h*.

(1) $$\begin{array}{r} u=\frac{l_{tendon\;at\;0^{{}^{\circ}}}-h}{radius} \end{array}$$

(2) $$\begin{array}{r} v=\frac{-h+l_{tendon\;at\;-90^{{}^{\circ}}}}{radius} \end{array}$$

As defined in Allen et al. (2020) *w* is defined as [*u, v*, 0] and whose magnitude equals *ϕ*. Therefore, *ϕ* represents the total magnitude of the deflection angle as shown in Equation (3)

(3) $$\begin{array}{r} \phi =\sqrt{u^{2}+v^{2}} \end{array}$$

The full homogeneous transformation matrix for the *uvh* parametrization is described in Allen et al. (2020). We use this to compute the position of the end of each link along the kinematic chain of segments that makes up the complete pose of the bending section of the continuum joint.

This approach is used for each estimation method described in section 2.5. Although each sensor covers several constant curvature segments, these segments may not have the same curvature. Thus at least some error is introduced. The tendon length *l* of a segment is calculated by dividing the full sensor by the number of segments that it covers. This tendon length is referred to as a “virtual tendon length.”

Given every segment's angle of deflection the length of a simulated sensor is calculated by summing the “virtual tendon lengths” for each segment that the sensor covers. The “virtual tendon lengths” are calculated by solving for the respective *l* found in Equations (1) and (2).

### 2.4. Loading Conditions

**Figure 5:**
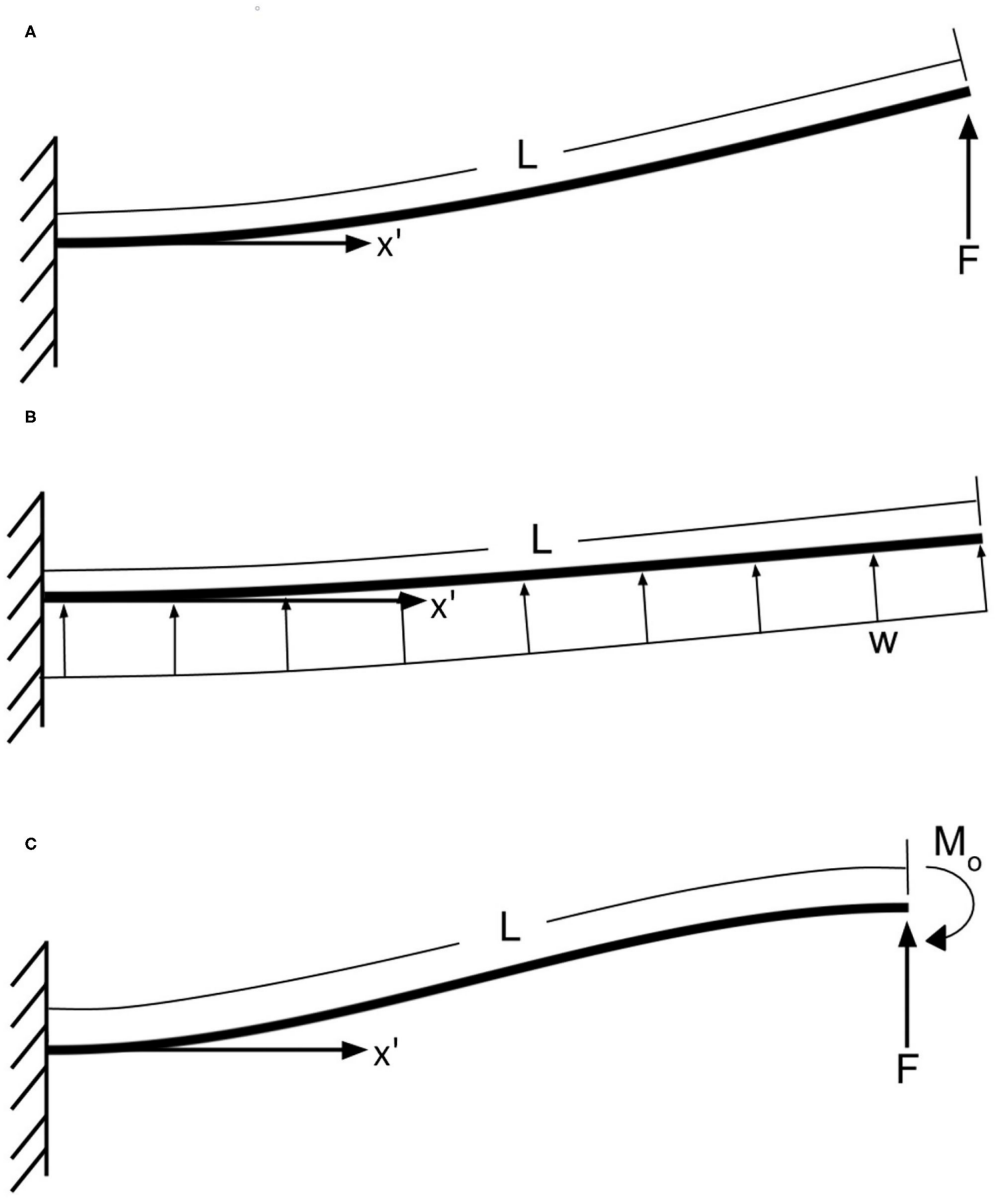
**(A)** End force load, **(B)** uniformly distributed load, **(C)** beam with one end fixed and the other end guided. *L* is the arc length of the full joint, *F* is an end load amount, *w* is a distributed load, and *M*_0_ is a moment.

Since the motivation of this paper is to improve the estimation of continuum joint poses under real-world loading conditions, we examine four loading conditions that encapsulate the majority of situations experienced by a cantilevered continuum joint with a fixed mounting. The loading conditions are listed as follows:

- **End Force Load**: This loading condition simulates contact at the end of the joint (Figure 5A).
- **Uniformly Distributed Load**: This loading condition simulates joint deflection due to gravity (Figure 5B).
- **End Force Load with a Moment**: This loading condition simulates a load at the end of the joint with a torque created by the joints actuators resulting in an s shape (Figure 5C).
- **Constant Curvature**: While not explicitly a loading condition, this represents the joint being actuated such that all segments reach their maximum range of motion (ROM).

We treat the continuum joint as a cantilevered beam and apply each of the given loading conditions. The Finite Element Analysis (FEA) program ANSYS was used to simulate the resulting deformation of the modeled beam.

We use Beam 188 elements which were divided into *m* * 3 elements, where *m* is the number of the constant curvature segments in the joint. For each loading condition the load and/or moment was incrementally increased until the desired deflection of the first segment was reached.

It is important to note that we used the FEA solution for all of the loading except for the constant curvature case as the angles of deflection for each segment are all the same and thus known.

We then adapt these nonconstant curvature simulations to our actual Piecewise Constant Curvature (PCC) joint model. This is done by recording the total deflections from the FEA model at the beginning and end of each segment. Then the difference between the deflection at the beginning of a segment and the end of the segment is calculated. This difference is then set as the bending angle for that Constant Curvature segment as shown in Equation (4).

(4) $$\begin{array}{r} \phi_{i}=\theta_{i+1}-\theta_{i}\forall i=1,\ldots ,m \end{array}$$

**Figure 6:**
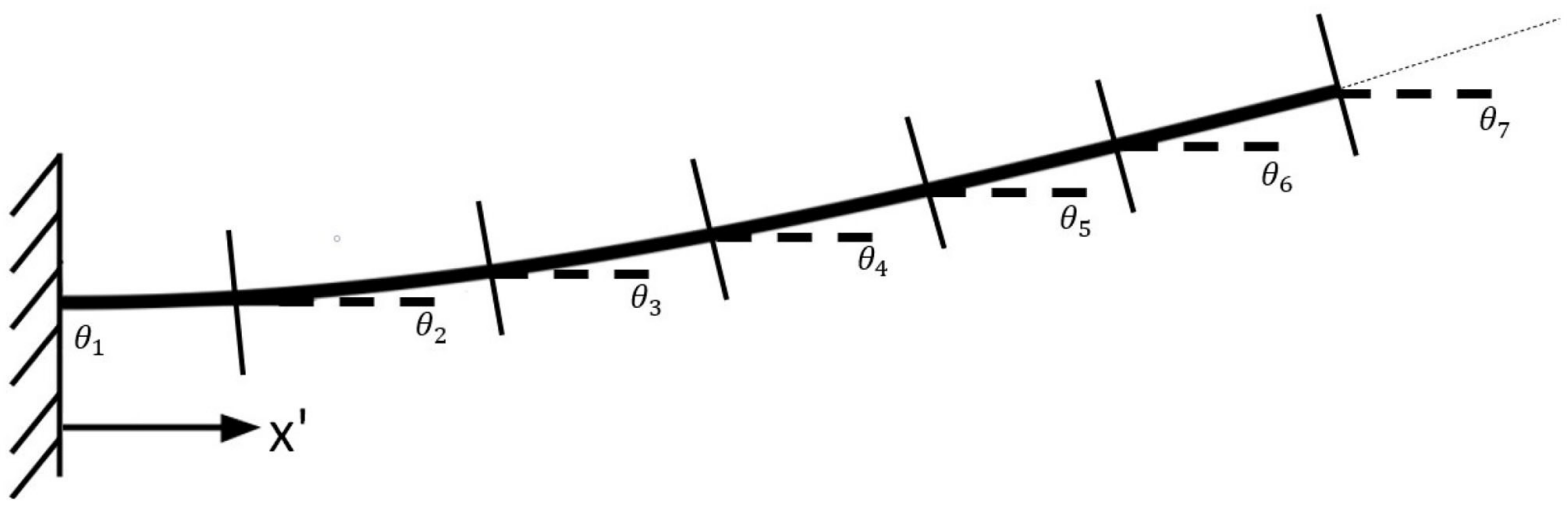
Illustration of the method used for adapting a continuous bending model to a Piece-wise Constant Curvature model.

Figure 6 demonstrates a continuum joint undergoing a force load and the deflection angles, *θ*_*i*_ that can be used to calculate the relevant joint angles *ϕ*_*i*_

Due to the computational demands of the finite element analysis (FEA), we found solutions for a specific set of joint deflections. This was performed for every loading condition where the first segment was set to the maximum ROM which was incremented from −8 to +8° in increments of ${\frac{1}{8}}^{{}^{\circ}}$. For any modeled deflections that were between the original FEA solutions a linear interpolation was used. Using this method, a maximum per segment error bound of 0.0285° was calculated for the linear interpolation. This was calculated using the worst case scenario (maximum difference between two points used for interpolation) in terms of error. Specifically, the error bound was found by summing the difference between joint angle FEA solutions used for interpolation and then dividing by the number of segments. We did not need to use the interpolation method for the constant curvature loading case as each segment's angle would be the maximum ROM.

### 2.5. Estimation Methods

In this section we describe the estimation methods used in the simulated experiments. We experimented with simulating four different sensor configurations for gathering state data and used two different methods for state estimation.

#### 2.5.1. Sensor Configurations

When describing the configuration of sensors along the length of a continuum joint, we use a pair of two numbers inside square brackets to represent the sensor's starting segment and ending segment as such [starting segment–ending segment]. Figure 4 shows the indexing of the joint segments on this 12 segment continuum joint. The segment numbering is started at the most proximal segment which is labeled segment 0 and the rest of the segments are incrementally labeled until the last segment. Using our method of describing a sensor configuration on a joint, the red sensor is [0–11], the blue sensor is [1–3], and the green sensor is [6–8].

##### 2.5.1.1. Single Sensor

The Single Sensor configuration, henceforth abbreviated as SS, involves using a single sensor that spans the entire length of a joint, to measure the overall joint angle of a continuum joint. This method relies on the assumption of constant curvature along the entire length of the joint for state estimation. This method represents the bare minimum amount of sensing that a continuum joint can have for state estimation with length sensors.

##### 2.5.1.2. End-to-End

The End-to-End Sensor configuration, henceforth abbreviated as EE, involves multiple sensors that are placed along the length of the joint with every segment covered and no overlap. The method for algorithmically determining the sensor placement involves dividing the number of segments by the number of sensors and rounding down. That is the default number of segments each sensor will cover. If there is a remainder from dividing the number of segments by the number of sensors, that remainder is evenly distributed among the sensors closest to the distal end of the joint. For example, a 12 segment joint with five sensors would have sensors that cover the following segments [0–1], [2–3], [4–5], [6–8], and [9–11].

##### 2.5.1.3. Heuristic Overlap

The Heuristic Overlap configuration, henceforth abbreviated as HO, involves multiple sensors aligned in a regular pattern along the joint, with each sensor overlapping with its neighboring sensors for two segments. The sensor placement is determined by first finding the EE sensor configuration and expanding each sensor's starting and ending index by one segment. Note, sensors that cover the first or last segment on the joint are not expanded past the ends of the joint. For example, a 12 segment joint with five sensors would have sensors that cover the following segments [0–2], [1–4], [3–6], [5–9], and [8–11].

##### 2.5.1.4. Optimized Overlap

The Optimized Overlap configuration, henceforth abbreviated as OO, is a sensor configuration that is determined by an evolutionary algorithm we developed. The evolutionary algorithm is described in section 2.6. This configuration represents the best possible sensor configuration for a given number of sensors.

#### 2.5.2. State Estimation Methods

For each estimation method, we estimate the angle of deflection for each individual segment of the continuum joint which then allows for the estimation of the full configuration of the joint. This is accomplished by estimating the length that a single sensor would be if it was monitoring just that individual segment, henceforth known as the“virtual sensor length.” We have developed three methods for performing this estimation.

##### 2.5.2.1. DEM (Direct Estimation Method)

For sensor configurations that have no overlap, we estimate the virtual sensor length of a segment by simply dividing the length of the sensor covering it by the number of segments that sensor covers. This method assumes that all of the segments covered by a sensor have the same angle of deflection.

##### 2.5.2.2. E-WAM (Equally-Weighted Averaging Method)

For sensor configurations that have overlap, we have two methods of estimating the virtual sensor length of a joint segment, the first of which we call E-WAM. The E-WAM method takes the per segment lengths of all the sensors covering a segment and averages them to estimate the length of the virtual sensor for that segment.

##### 2.5.2.3. WAM (Weighted Averaging Method)

The second method for estimating the virtual sensor length, *l*_*est*_, of a joint segment on a robot that has overlapping sensors is WAM. This method uses a weighted linear combination of all of the sensors on the robot to find the virtual sensor length for each segment, the hypothesis being that the sensors that do not cover the segment still provide additional information about its state. Each segment has a separate weight for each sensor on the joint as shown in Equation (5).

(5) $$\begin{array}{r} l_{est,i}=\overset{n}{\underset{j=0}{\sum }}w_{i,j}*\frac{l_{j}}{p_{j}} \end{array}$$

where *i* is the *ith* segment, *n* is the number of sensors on a joint, *w*_*i,j*_ is the weighting on the *ith* segment for the *jth* sensor, *l*_*j*_ is the full length of the *jth* sensor, and *p*_*j*_ is the number of segments the *jth* sensor spans.

We find these weights by applying the robust linear regression algorithm from Scipy (Virtanen et al., 2020) to deflection angle data we simulated from the continuum joint under 30 different loading samples (*s*) for each of the 4 loading conditions (*c*) for a total of 120 data points per joint segment. The 30 different loading samples are calculated by varying the ROM used in the loading conditions as describe in the following equation.

(6) $$\begin{array}{r} ROM_{loading,i}=-ROM_{max}+i\frac{2ROM_{max}}{s}\forall i=1,\ldots ,s \end{array}$$

We use the scipy *least_squares* function with the loss condition set to “*soft_l1*” and the “*f_scale*” condition set to 0.1. Our residuals function can be seen in Equation (7) where *S* is the matrix of collected sensor data, *w*_*i*_ is a vector of the weights for which we are solving, and *l*_*i*_ is the length of a virtual sensor covering that segment.

(7) $$\begin{array}{r} residual=Sw-l_{i} \end{array}$$

Matrix *S* takes the form shown in Equation (8). Each row is made up of the sensor values from one of the simulated loading cases. The sensor data in the matrix is normalized and denoted as $\bar{s}$, where ${\bar{s}}_{j}=\frac{l_{j}}{p_{j}}$.

(8) $$\begin{array}{r} S=\left[\begin{array}{cccc} {\bar{s}}_{1,case\;1} & {\bar{s}}_{2,case\;1} & \ldots & {\bar{s}}_{n,case\;1}\\ {\bar{s}}_{1,case\;2} & {\bar{s}}_{2,case\;2} & \ldots & {\bar{s}}_{n,case\;2}\\ \vdots & \vdots & \ddots & \vdots \\ {\bar{s}}_{1,case\;m} & {\bar{s}}_{2,case\;m} & \ldots & {\bar{s}}_{n,case\;m} \end{array}\right] \end{array}$$

Vector *w*_*i*_ takes the form shown in Equation (9).

(9) $$\begin{array}{r} w_{i}=\left[\begin{array}{c} w_{i,sensor\;1}\\ w_{i,sensor\;2}\\ \vdots \\ w_{i,sensor\;n} \end{array}\right] \end{array}$$

Vector *l*_*i*_ takes the form seen in Equation (10).

(10) $$\begin{array}{r} l_{i}=\left[\begin{array}{c} l_{i,case\;1}\\ l_{i,case\;2}\\ \vdots \\ l_{i,case\;m} \end{array}\right] \end{array}$$

### 2.6. Evolutionary Algorithm

To find the optimal sensor placement on a continuum joint, we implemented an Evolutionary Algorithm (EA) from the DEAP (Distributed Evolutionary Algorithms in Python) Library (Fortin et al., 2012).

The goal of our algorithm is to find the optimized sensor placement for a given continuum joint with a fixed number of sensors. Prior to running the EA, we define the continuum joint on which we will be optimizing the sensor placement by setting the total length of the joint, the number of segments, and the total range of motion of the continuum joint.

The EA itself is the eaSimple function from the DEAP library, which handles iterating over the specified number of generations, selection, mating and mutation with built-in options or the ability to define your own functions. We chose to do 10 generations and discuss our choices for selection, mating, and mutation in section 2.6.4.

#### 2.6.1. Defining an Individual

To represent an individual we used a list of integers with a length of two times the number of sensors. For example, a continuum joint with 12 segments and two sensors could be represented as [0, 7, 4, 11]. In this list, each sensor is represented by a pair of numbers. The first two numbers represent the starting segment index of the first sensor and the ending segment index of the first sensor. The second two numbers represent the starting and ending index of the second sensor. For a given sensor number *i*, the starting index is 2*i* and the ending index is 2*i*+1. If the ending index is lower than the starting index, they are automatically swapped to be in the correct order by our algorithm. The sensors cover the full segments of both the starting and ending segment. In other words the sensor starts on the bottom of the starting segment and ends at the top of the end segment.

#### 2.6.2. Creating the Population

To create the population, we create 500 individuals each with an attribute list that is two times the number of sensors long with random integers generated at every index of the attribute list.

We experimented with seeding the population with individuals that have sensors lined up end to end or start with a Heuristic overlap but found no noticeable improvement in the EA's performance.

#### 2.6.3. Evaluating the Fitness

To evaluate the fitness of an individual we use a cost function that sums the deflection angle error of all *m* joint segments, for all *s* loading samples of a loading conditions, for all *c* loading conditions giving us the cost function seen in Equation (11). Our goal is to minimize the cost of an individual.

(11) $$\begin{array}{r} cost=\overset{c}{\underset{i}{\sum }}\overset{s}{\underset{j}{\sum }}\overset{m}{\underset{k}{\sum }}\left(\left|\phi_{actual,i,j,k}-\phi_{estimated,i,j,k}\right|\right) \end{array}$$

Additionally, when evaluating an individual, we first determine whether or not a sensor configuration is a valid configuration. For our purposes, valid means that each segment on the joint is observable i.e., covered by at least one sensor. If this criteria is not met, the individual's fitness score is set to the maximum which is the maximum joint error possible [(2×*ROM*) multiplied by *c*, *s*, and *m*]. Intuitively, this means that the estimation was off by the maximum possible amount for each segment in each loading simulated.

We also experimented with including the Cartesian position and orientation of the end effector of the joint in the fitness score. However, due to its direct correlation with the individual deflection angles we found that this did not improve overall performance for the optimization.

#### 2.6.4. Selection, Mating, and Mutation

Selection is performed though a tournament selection process as provided by the DEAP library, *deap.tools.selTournament(individuals, k, tournsize, fit_attr=“fitness”)*, where the method is passed a list of individuals (individuals) and the size of the tournament (tournsize).

The mating is performed by using a one point crossover algorithm provided by the DEAP library, *deap.tools.cxOnePoint(ind1, ind2)*, where “*ind1*” and “*ind2*” are two individuals that are to be mated. The algorithm randomly chooses a place for crossover to happen. Crossover then occurs by swapping the elements between the two individuals that are right of the selected element. This method cannot choose the last element so there will always be some crossover. We set the crossover probability to 0.7.

Mutation occurs using the method *deap.tools.mutUniformInt(individual, low, up, indpb)* found in the DEAP library where “*individual*” is the individual to be mutated, “*low*” and “*up*” are the lower and upper bound, respectively, that an attribute can be set to, and “*indpb*” is the independent probability that each element of the attribute will be mutated. Therefore, if an individual is selected for mutation each element of the individual's attribute (the sensor list) has a chance to randomly mutate to a value in the closed set [“*low*”, “*up*”] based on a uniform distribution. We set the mutation probability to 0.5

### 2.7. Experiments

We had four hypotheses that we tested and analyzed for general trends.

1. Increasing the number of sensors on a joint for a given placement method and estimation method will improve the accuracy of the state estimation.
2. Overlapping the sensors can provide more information about the configuration of the joint and will therefore improve configuration estimation for continuum joints.
3. Using a weighted linear combination of the overlapping sensor data can decrease the state variable estimation as compared to an equally weighted linear combination. Additionally, the weights can be found using linear regression.
4. An evolutionary algorithm can be used to determine the optimal placement of overlapping sensors that will further improve state estimation for continuum joints.

To prove generality of our solutions and to test the hypotheses being proposed, we generated 80 different joints by varying the number of segments and the max ROM per segment. We varied the two variables as shown in Table 1 to generate the 80 different joints. From here on in this paper, when we mention ROM, we are referring to the range of motion of the segment, not of the whole joint, unless explicitly stated otherwise.

**Table 1:** The different continuum joint parameters and their values that were simulated.

**Parameters**	**Values**
Number of segments	12, 16, 20, 24, 28, 32, 36, 40, 44, 48
Max ROM per segment (degrees)	±1, ±2, ±3, ±4, ±5, ±6, ±7, ±8

For each of the hypotheses presented above, we perform simulated experiments that compare the performance of a sensor placement or state estimation method on all 80 joints. We compare the performance of the methods by simulating the joints in 40 different poses and comparing the aggregate error of the joint segments angle error (our cost function) normalized for number of segments *m*, max ROM, loading conditions *c*, and loading samples, *s*. The 40 different poses come from the 4 different loading cases (*c* = 4) and ten sample poses (*s* = 10). We then multiply by 100 to get the average joint segment angle error as a percent of ROM for a given joint (see Equation 12).

(12) $$\begin{array}{r} Average\;\%\;Error\;of\;ROM=\frac{cost}{c\cdot s\cdot m\cdot ROM_{max}} \end{array}$$

With the exception of the base case of a single sensor, we performed all of our simulated experiments with two, three, four, five, and six sensors to study how the results change as more sensors are added. For the first hypothesis, we compare the average percent error of ROM when using the DEM on the simulated joints for the SS placement and two to six sensors in the EE placement. For the second hypothesis we study the effects of overlap by comparing the results of the EE placement method with the DEM estimation method vs. the HO placement method with the E-WAM estimation method. The third hypothesis tests our WAM method by comparing the WAM method and the E-WAM method on the joints with HO sensor placement. The final hypothesis tests our evolutionary algorithm by comparing the HO and OO sensor placement methods while using the WAM estimation method. Section 3 presents and discusses the results of the simulated experiments.

## 3. Results

**Figure 7:**
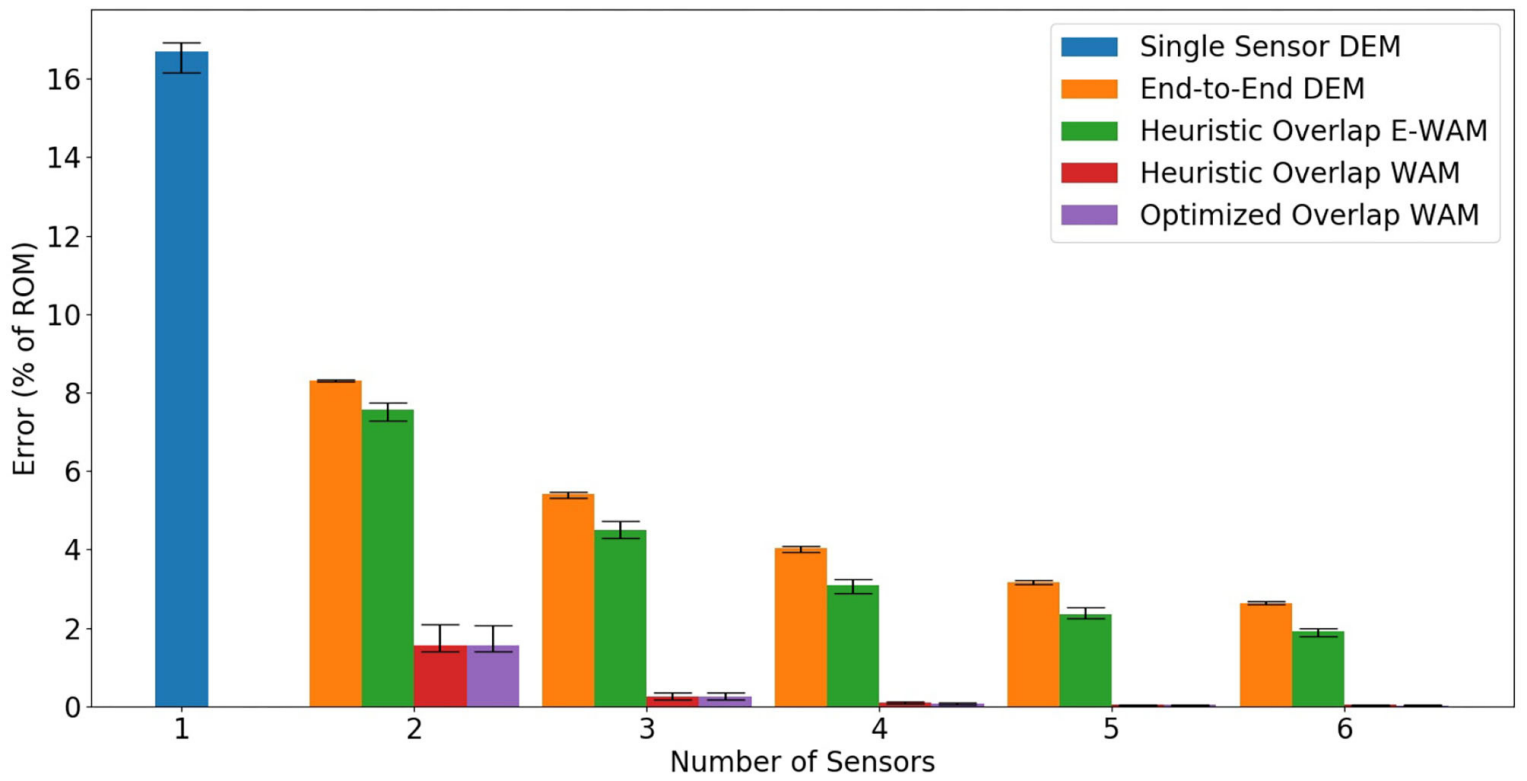
This bar graph shows the median results for all of the simulated joints' “Average % Error of ROM” (defined in Equation 12) of all the joint's segments. Quartile bars are included to show the spread of the results.

This section reports the results of the tests described in section 2.7. To make it easier to compare all of our results, we created a bar graph that summarizes the tested sensor placement and estimation methods, shown in Figure 7. We also report the original data used to generate the bar graph in Table 2.

**Table 2:** Average segment error as a percent of the range of motion, normalized over all of the deflection modes used for evaluation of performance.

	**# Sensors**	**1**	**2**	**3**	**4**	**5**	**6**
	Median	16.71	–	–	–	–	–
SS, DEM	3rd Quart.	16.92	–	–	–	–	–
	1st Quart.	16.16	–	–	–	–	–
	Median	–	8.309	5.423	4.033	3.178	2.634
EE, DEM	3rd Quart.	–	8.321	5.465	4.077	3.212	2.672
	1st Quart.	–	8.281	5.305	3.931	3.105	2.592
	Median	–	7.561	4.504	3.090	2.342	1.914
HO, E-WAM	3rd Quart.	–	7.735	4.720	3.230	2.538	1.993
	1st Quart.	–	7.277	4.280	2.878	2.257	1.784
	Median	–	1.552	0.262	0.0915	0.0434	0.0366
HO, WAM	3rd Quart.	–	2.086	0.364	0.112	0.0538	0.0475
	1st Quart.	–	1.403	0.181	0.0653	0.0289	0.0228
	Median	–	1.542	0.238	0.0836	0.0368	0.0315
OO, WAM	3rd Quart.	–	2.063	0.362	0.106	0.0506	0.0437
	1st Quart.	–	1.389	0.175	0.0427	0.0220	0.0215

*SS, single sensor; EE, end to end; HO, heuristic overlapping; OO, optimized overlapping; DEM, direct estimation method; E-WAM, non-weighted averaging method; WAM, weighted averaging method*.

**Figure 8:**
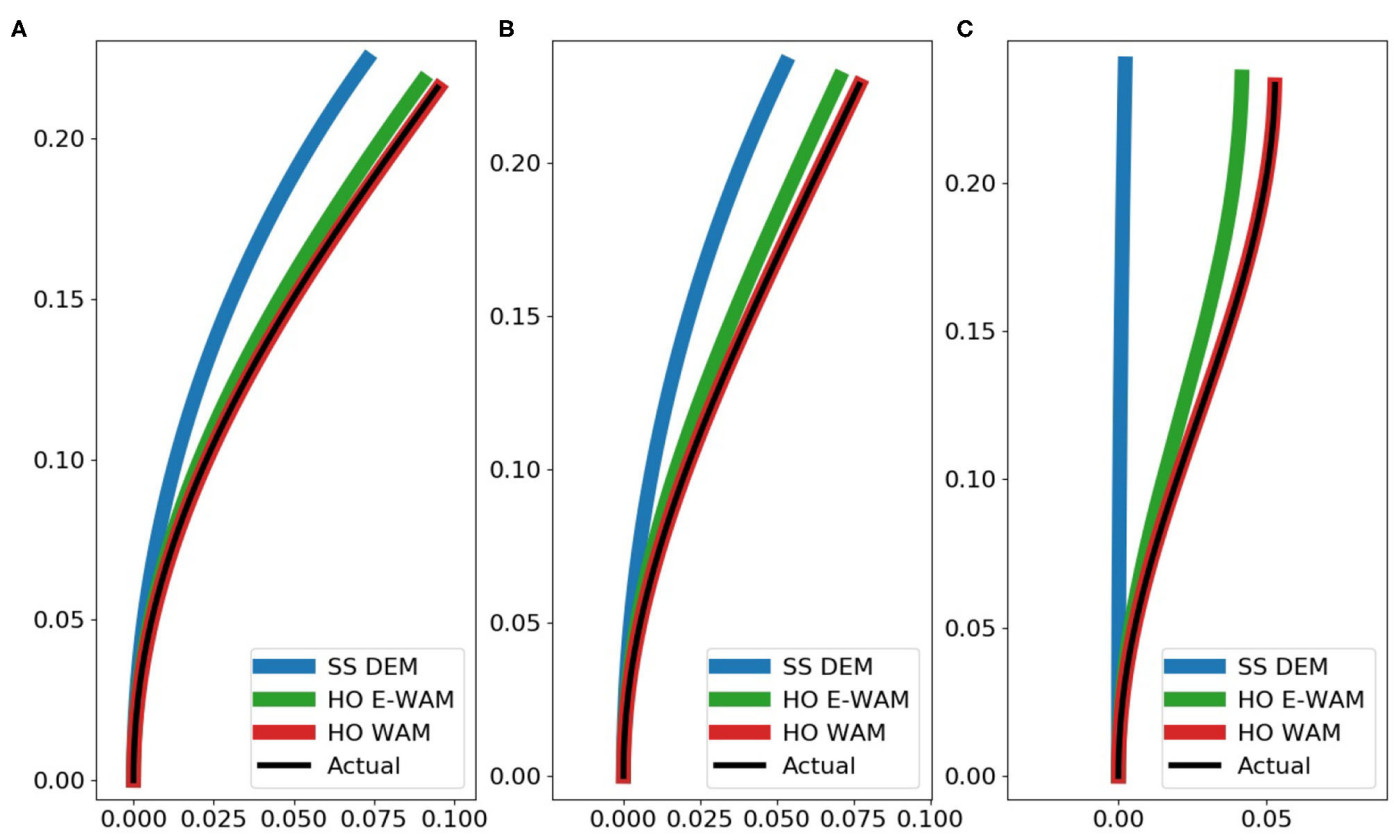
Case study of effectiveness of estimation methods for a single case. Sensors placed using HO. Number of segments = 12, max ROM = 3 deg, and number of sensors = 3 placed at [0,4,3,8,7,11]. The plots show the following loading conditions: **(A)** end force load, **(B)** uniformly distributed load, **(C)** moment and end force load. All units in the plots are reported in meters.

We have also included a case study to help visually show the effectiveness of the different estimation methods for the different loading cases. Figure 8 shows how well different estimation methods are able to reconstruct the actual configuration of the joint under the three real-world loading scases.

## 4. Discussion

The results of these simulated experiments strongly support our first, second, and third hypotheses. The results also loosely support our fourth hypothesis. For all of the analyses listed in this discussion, the data behind Figure 7 can be found in Table 2.

Our first hypothesis, that increasing the number of sensors on a joint for a given placement and estimation method will improve the accuracy of the state estimation, is somewhat intuitive. As we can see in Figure 7, as the number of sensors increases, the error, as a percent of the range of motion, generally decreases. This hypothesis is most strongly supported by the EE with DEM and HO with E-WAM state estimation methods. For these methods, the decrease in error resembles an exponential decay. While six sensors was the maximum number of sensors we used in our experiments, we expect the decrease in error for a given continuum joint to plateau when the number of sensors is greater than or equal to the number of segments. For example, a twelve segment joint using EE and DEM with 13 sensors would not be any more accurate than a joint with twelve sensors given our assumptions. In the real world, with imperfect sensors, this may not be true because having two sensors monitoring a single segment may allow filtering or averaging to get more accurate information out of the two sensors than a single sensor alone.

While the trend of increasing the number of sensors leading to a decrease in error is consistent across all the simulated experiments, we also noticed a steep decline in error for HO and OO using the WAM method when going from two sensors to three sensors, with an effective plateau in performance from four to six sensors. We attribute this plateau to the effectiveness of the WAM method to accurately estimate the state with a smaller number of sensors. Four or more sensors seems to add redundant information to the estimation method resulting in only minor decreases in error.

Our second hypothesis states that by overlapping length sensors on a continuum joint, we are able to obtain more information about its configuration and therefore better estimate said configuration. Referencing Figure 7 again, we can see that all cases of HO or OO had lower errors than the EE placement method for a given number of sensors. This confirms that overlapping sensors does indeed allow us to more accurately estimate the configuration of the joint.

We first analyze why there is an improvement from using EE with DEM to HO with E-WAM. This is performed using the term “region of estimation,” which refers to groups of segments on the continuum joint which are estimated to have the same deflection angle and therefore the same curvature. In a simplified example, a continuum joint with two sensors with EE placement only has two distinct regions of estimation, the segments covered by the first sensor and the segments covered by the second sensor. A continuum joint with two sensors using the HO placement has three distinct regions of estimation, the segments covered exclusively by the first sensor, the segments covered exclusively by the second sensor and the segments covered by both sensors. The E-WAM method is essentially the DEM method but it averages the overlapping sensors that are covering a segment. This creation of additional estimation regions is what allows the HO method to have lower error than the EE method, even when using a simple estimation method such as E-WAM.

Our third hypothesis, which is the main contribution of this paper, is that a weighted linear combination of overlapping sensor data can significantly reduce state estimation error when compared to simpler estimation methods such as E-WAM and DEM. The reduction in error from HO with E-WAM to HO with WAM shown in Figures 7, 8 is dramatic. This data is highlighted in Table 3. We can easily see how overlapping sensors creates additional regions of estimation with the simple estimation method E-WAM. The WAM method takes that one step further by using linear regression to derive unique sensor value weights for estimating the state of each segment, thus creating a distinct region of estimation for each segment. This means that each joint segment can have a unique estimated deflection angle with minimum of two sensor. To achieve this with E-WAM *m* − 1 sensors are needed, where *m* is the number of constant curvature segments of the joint. For example, the proximal most segment is always bent at an angle that is greater than or equal to the bending angle of the next most proximal segment. This can be expressed by the WAM method when it calculates slightly different weights for segments zero and one, even though they may be covered by the same set of sensors.

**Table 3:** Table highlighting the difference in median error as a % of ROM between HO with E-WAM and HO with WAM.

**Number of sensors**	**2**	**3**	**4**	**5**	**6**
HO with E-WAM median error as % of ROM	7.561	4.504	3.090	2.342	1.914
HO with WAM median error as % of ROM	1.552	0.261	0.0915	0.0434	0.0366
**Decrease in median error as % of ROM**	**6.009**	**4.243**	**2.9985**	**2.2986**	**1.8774**

*The bold values shown in the table highlight the improvements between the two methods being compared*.

Furthermore, the WAM method allows for sensors that are not covering a segment to provide information about the robot state. By using a linear combination of all the sensor measurements on the joint, not just the ones covering the segment, WAM is able to significantly reduce the deflection angle estimation error as compared to E-WAM. For example, if the proximal segments have a sensor reading associated with a negative bending angle and the distal segments have a sensor reading associated with a positive bending angle, that information can be captured by the weights of the WAM method to determine that there will be a point of inflection in the middle of the joint and therefore middle segments will have small deflection angles in this situation.

Our final hypothesis was that an evolutionary algorithm could be used to determine the optimal placement of overlapping sensors such that state estimation will be further improved than using WAM with the Heuristic Overlap. This hypothesis is only loosely supported by the data collected in our simulated experiments. Since the bars in  Figure 7 are so small, the data comparing HO and OO with WAM are highlighted in Table 4. There is always a reduction in error when using OO instead of HO, however that reduction is very small. We mainly attribute this to the WAM method being able to estimate the shape so accurately that it is difficult to reduce the error even further using “optimal” sensor placements. We also believe the HO placement method already provides a fairly optimal, even coverage of all the segments on the joint. The largest reduction in error observed occurs when using three sensors. We believe the benefits of OO peak at three sensors because it is when using more than three sensors with HO there is already excellent coverage of the segments and when using two sensors there aren't many possible configurations so there is only a modest reduction in error from optimizing.

**Table 4:** Table highlighting the difference in median error as a % of ROM between HO with WAM and OO with WAM.

**Number of sensors**	**2**	**3**	**4**	**5**	**6**
HO with WAM median error as % of ROM	1.552	0.262	0.0915	0.0434	0.0366
OO with WAM median error as % of ROM	1.542	0.238	0.0836	0.0368	0.0315
**Decrease in median error as % of ROM**	**0.010**	**0.0240**	**0.00790**	**0.0066**	**0.0051**

*The bold values shown in the table highlight the improvements between the two methods being compared*.

In conclusion, we have shown that state estimation of a continuum joint can be significantly improved by using the WAM estimation method on overlapping sensors which are placed on the continuum joint according to a simple heuristic. Using this method with only three sensors yielded a median joint angle error (as a percentage of the range of motion) of 0.262%. Increasing the number of sensors further reduced the state estimation error to under 0.1%. We have also shown that the simple heuristic overlap performs almost as well as an optimized overlapping arrangement determined by an evolutionary algorithm with the median error (as a percent range of motion), being <0.025% for all cases tested.

Some sources of error in this work could come from the shapes of the joints in the real world not being as ideal as the simulated ones we used for testing. This would mean that the median errors determined in this paper would be slightly higher when implemented on hardware even with ideal sensors. Even with this introduction of uncertainty, we are confident the reduction in error seen from using WAM in simulation will translate to large, real world reductions in error. A simple way to improve the estimation would be to collect test data from the hardware and perform linear regression on that data rather than simulated data. Nonetheless, future work will entail implementing these sensor placement and configuration estimation methods on hardware and testing their capabilities for a non-idealized sensor. Given noise or other possible sources of error introduced by the hardware, this will be important to prove that the approach is as effective in the real world as is predicted by these kinematic and static loading simulations.

## Data Availability Statement

The raw data supporting the conclusions of this article will be made available by the authors, without undue reservation.

## Author Contributions

LR and TD developed the concepts presented here with support from MK. All authors contributed equally to the manuscript.

## Conflict of Interest

TD is employed by company Otherlab Inc., while LR was formerly employed by company Otherlab Inc. The remaining authors declare that the research was conducted in the absence of any commercial or financial relationships that could be construed as a potential conflict of interest.
